# Trans-Pfannenstiel sigmoidectomy for sigmoid volvulus: description of a novel surgical technique and initial experience from a retrospective case series

**DOI:** 10.1007/s00384-026-05143-5

**Published:** 2026-05-07

**Authors:** Valentina Murzi, Mauro Podda, Francesco Balestra, Marcello Pisano, Alessandra Saba, Alessia Dessì, Raimondo Sanna, Eleonora Silanos, Marco Puledda, Adolfo Pisanu

**Affiliations:** 1 Department of Surgical Science, University of Cagliari, Cagliari, Italy; 2 Department of Surgery, General Surgery Unit, Policlinico Sassarese, Sassari, Italy; 3 Department of Emergency, Emergency Surgery Unit, Policlinico Universitario Di Monserrato, Azienda Ospedaliera-Universitaria Di Cagliari, Cagliari, Italy

**Keywords:** Sigmoid volvulus, Sigmoidectomy, Pfannenstiel incision, Colorectal surgery, Elderly patients, Frailty

## Abstract

**Purpose:**

Sigmoid volvulus is a recurrent cause of large bowel obstruction that predominantly affects elderly and frail patients. After successful endoscopic detorsion, elective sigmoid resection is recommended to prevent recurrence. This study describes a new, trans-Pfannenstiel approach for sigmoidectomy and reports the initial clinical experience with this technique.

**Methods:**

This study was designed as a single-center retrospective case series. Adult patients surgically treated for sigmoid volvulus between 2024 and 2025 were included. All patients underwent successful endoscopic detorsion and decompression followed by planned surgical resection. The primary outcome was postoperative complications within 30 days. Surgical technique, perioperative outcomes and short-term follow-up were analyzed.

**Results:**

Eleven patients were included, with a median age of 71 years (IQR 51–79); five patients (45.4%) were classified as ASA III, and nine patients (63.6%) had experienced two or more previous episodes of volvulus. Median operative time was 105 min (IQR 90–125). No patient required postoperative intensive care or reoperation. Postoperative complications occurred in four patients (36.4%), with one Clavien-Dindo grade IIIa complication managed non-operatively with CT-guided percutaneous drainage. Median length of hospital stay was 6 days (IQR 5–6). Three patients (27.3%) required early readmission for medical complications (one Clavien-Dindo IIIa and two Clavien-Dindo II complications). No postoperative mortality or recurrence of sigmoid volvulus was observed during a median follow-up of 394 days (IQR 246–434).

**Conclusions:**

Trans-Pfannenstiel sigmoidectomy is a feasible, safe, and reproducible technique for the surgical management of sigmoid volvulus in selected patients. When performed after endoscopic decompression in a planned setting, it allows definitive treatment while limiting abdominal wall trauma in a fragile population.

**Supplementary Information:**

The online version contains supplementary material available at 10.1007/s00384-026-05143-5.

## Introduction

Sigmoid volvulus (SV) is an uncommon cause of large bowel obstruction, resulting from axial rotation of a redundant sigmoid colon around an elongated mesentery with a narrow base [1]. SV accounts for 2–3% of intestinal obstructions in Western countries. Its incidence is higher in Africa, Middle East, South America, and Asia, where it may represent up to 10–50% of cases [2, 3]. SV predominantly affects elderly patients and is frequently associated with chronic constipation, neurological or psychiatric disorders, and institutionalization. Morbidity and mortality increase in the presence of bowel ischemia or perforation [4, 5].

In the absence of peritonitis or bowel necrosis, endoscopic detorsion with decompression is recommended as first-line treatment [6, 7]. Recurrence after endoscopic management alone is common, with reported rates ranging from 43 to 75%. Recurrent episodes expose patients to risks of bowel ischemia, perforation, clinical deterioration, and death [8–10]. For this reason, current international guidelines recommend elective sigmoid resection after successful detorsion, preferably during the index admission, including in elderly or high-risk patients [7, 11].

Although laparoscopy is widely used in elective colorectal surgery, its adoption in sigmoid volvulus remains limited. Data from the ACS NSQIP suggest that a laparoscopic approach is planned in only about 20% of cases, likely due to technical challenges related to colonic dilatation and limited maneuverability [12].

The excessive length and redundancy of the sigmoid mesentery may obscure the true axis of torsion, even for experienced laparoscopic surgeons, while elongated and often fibrotic mesenteries may harbor large, fragile vessels [11].

In this setting, a surgical approach that allows definitive resection while minimizing abdominal wall trauma may be advantageous. We describe a new technique of trans-Pfannenstiel sigmoidectomy for sigmoid volvulus and report our initial clinical experience, focusing on technical feasibility and short-term outcomes.

## Materials and methods

This study was designed as a single-center consecutive retrospective case series reporting the initial clinical experience with a low transverse Pfannenstiel incision for sigmoidectomy in patients with sigmoid volvulus. All adult patients undergoing surgical treatment for sigmoid volvulus between 2024 and 2025 at the Department of Emergency Surgery, University Hospital of Cagliari (Cagliari, Italy), were included. The study was conducted at a tertiary referral center serving a population of approximately 600,000 inhabitants.

All patients underwent preoperative endoscopic evaluation and decompression. Surgery was categorized as elective or planned during the index admission after successful endoscopic decompression (early elective). Patients requiring emergency surgery due to sigmoid ischemia or bowel necrosis, or extended multivisceral resections, were excluded. At our institution, the trans-Pfannenstiel approach is routinely adopted for patients undergoing elective or early elective resection after successful endoscopic decompression for primary sigmoid volvulus. No patients meeting the inclusion criteria during the study period were treated with alternative surgical approaches.

All procedures were performed by experienced emergency surgeons with expertise in both open and minimally invasive colorectal surgery.

All patients provided written informed consent for the surgical procedure, including authorization for intraoperative image and video recording for educational and research purposes. The study was conducted in accordance with the principles of the Declaration of Helsinki and reported in accordance with the PROCESS (Preferred Reporting Of Case Series in Surgery) guidelines [13].

The study was reviewed by the institutional Clinical Research Office of the University of Cagliari and the University Hospital of Cagliari. The requirement for formal ethical approval was waived according to local regulations.

### Clinical outcomes

The primary outcome of the study was postoperative complications. Secondary outcomes included operative data (operative time, associated procedures, and need for intensive care unit [ICU] admission), reoperations, length of hospital stay, time to nasogastric tube removal, bladder catheter removal, time to first bowel movement, resumption of oral intake, 30-day hospital readmissions, early and late recurrence of sigmoid volvulus, and late complications. Postoperative complications, readmissions, and reoperations were defined as events occurring within 30 days after surgery. Early recurrence of sigmoid volvulus was defined as recurrence within 30 days, while late recurrence was defined as recurrence occurring beyond 30 days. Late complications were defined as complications occurring more than 30 days after surgery. Follow-up was conducted according to a standardized protocol, including daily assessment during hospital stay, outpatient visits at 7 and 14 days after discharge, and telephone follow-up at 1 and 2 months. Patients were additionally contacted by telephone at the time of the study to assess late outcomes and recurrence.

### Data collection

Clinical data were collected retrospectively from a prospectively maintained institutional database. Variables were predefined before data extraction and included baseline characteristics (age, sex, body mass index, comorbidities, medications, previous abdominal surgery, ASA score, and prior episodes of sigmoid volvulus), clinical presentation (symptoms, presence of obstruction, timing of presentation and surgery, endoscopic decompression, and surgical setting), and diagnostic work-up (laboratory tests and imaging/endoscopic investigations).

### Statistical analysis

Continuous variables were reported as median and interquartile range (IQR), while categorical variables were expressed as absolute numbers and percentages. No comparative analyses or inferential statistical tests were performed. Statistical analyses were performed using *IBM SPSS Statistics version 30 (IBM Corp., Armonk, NY, USA).*

### Surgical technique

The procedure is routinely performed under general anesthesia. The operation may theoretically be performed under high thoracic regional anesthesia, provided that it is supported by an anesthesiology team with specific expertise in advanced loco-regional techniques [14–19].

The patient is placed in the supine position with the legs abducted. The upper limbs may be abducted or adducted according to the surgical team’s preference and anesthesiological requirements for venous access. The primary surgeon stands on the patient’s right side, with the first assistant positioned opposite the surgeon, while the scrub nurse with the instrument table is placed to the surgeon’s right. If not already in place preoperatively, a urinary catheter is inserted to prevent bladder distension and to facilitate safe construction of the anastomosis. Routine placement of a nasogastric tube is not required.

At hospital admission, a rectal tube is usually placed to decompress the sigmoid colon after endoscopic detorsion. The rectal tube may be removed at different time points: during patient positioning, after abdominal access and visualization of the sigmoid colon, or preoperatively within 24 h before surgery.

Preoperative removal may allow renewed sigmoid dilatation, facilitating intraoperative identification of the caliber transition between the sigmoid colon and the descending colon, which represents the planned site of colonic transection.

Skin preparation is performed using a chlorhexidine-based antiseptic, with antisepsis extending from the mammary line to the pubic region.

#### Surgical step 1: Surgical access and exposure (Video 1)

A Pfannenstiel incision is performed approximately two fingers above the pubic symphysis (about 10 cm in length). After transverse aponeurotic incision and separation of the rectus muscles, the peritoneal cavity is entered with attention to the urinary bladder. A dual-ring wound protector is positioned to optimize exposure.

The sigmoid colon is exteriorized, de-rotated, and repositioned. The proximal transition between the descending and sigmoid colon and the distal rectosigmoid junction are identified. Distal transection is planned below the rectosigmoid junction, avoiding an excessively low rectal division to preserve adequate stump length for a safe hand-sewn anastomosis.

#### Surgical step 2: Sigmoid resection (Video 2)

After identification of the proximal and distal transection points, sigmoid resection is performed, starting either proximally or distally according to surgeon preference. In the present video, resection begins at the rectosigmoid junction. The bowel is divided using a linear mechanical stapler after creation of a mesenteric window, followed by proximal transection at the predetermined site.

The mesentery is divided using an ultrasonic, radiofrequency, or combined ultrasonic-radiofrequency energy device close to the bowel wall to preserve vascular supply, as no oncological resection is required. In the presence of markedly thickened mesentery or enlarged sigmoid vessels, vascular control with clips may be advisable. The specimen is then removed and sent for histopathological examination.

#### Surgical step 3: Colorectal anastomosis (Video 3)

**Fig. 1:**
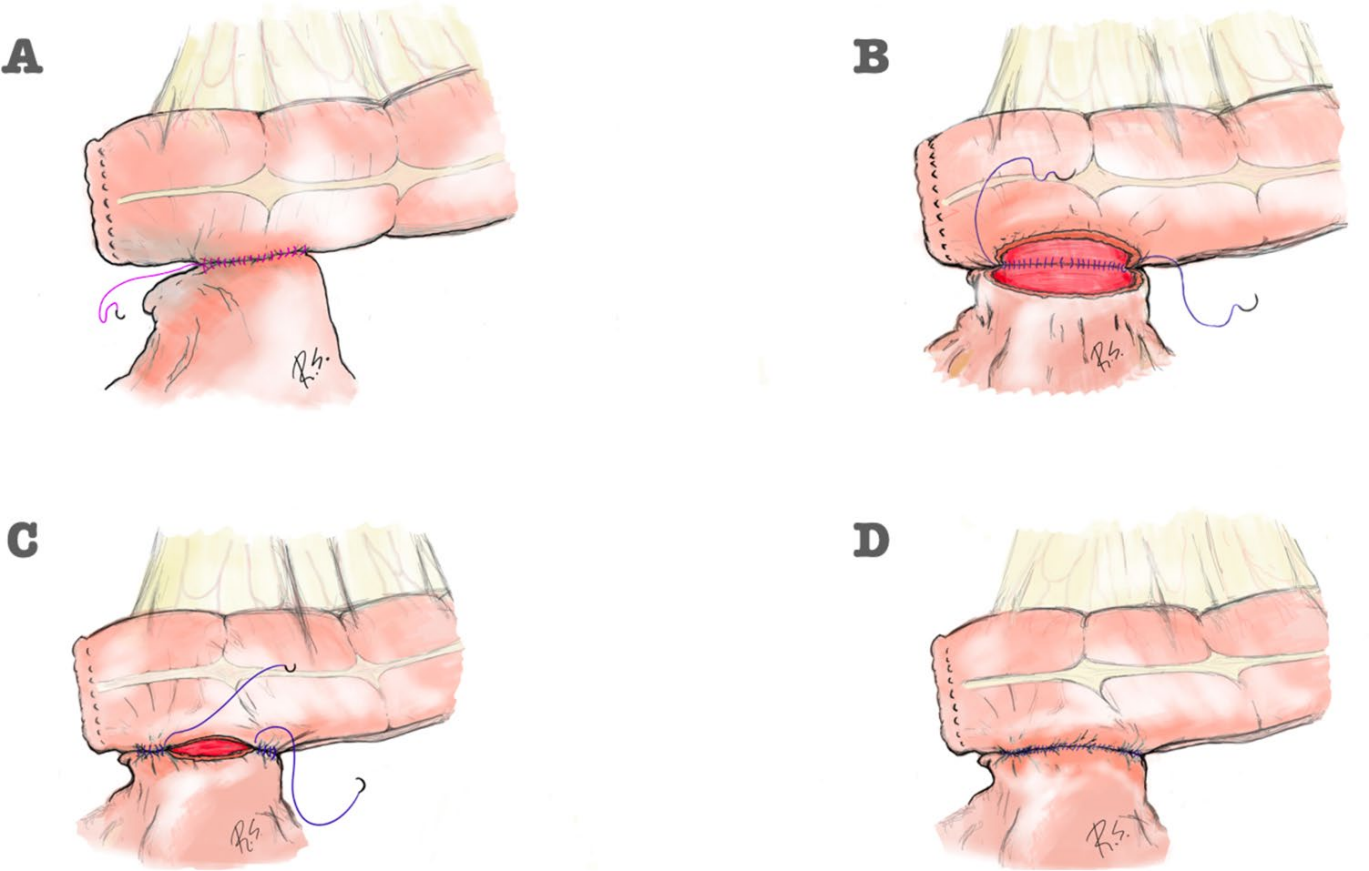
Hand-sewn side-to-end colorectal anastomosis. **A** Posterior approximation layer: the antimesenteric border of the colonic stump is approximated to the anterior wall of the rectal stump using a continuous 4-0 PDS suture. **B** Creation of colonic and rectal enterotomies followed by construction of the posterior anastomotic layer with a full-thickness or extramucosal 4-0 PDS suture, progressing towards and crossing the right angle. **C** Completion of the posterior layer towards the left angle and continuation of the anterior layer until the sutures meet and are tied at the center of the anastomosis. **D** Completion of the anterior reinforcing layer using a continuous or interrupted 4-0 PDS suture

A hand-sewn side-to-end colorectal anastomosis is routinely performed using a double-layer technique with 4-0 PDS sutures (Fig. 1A–D). When the calibers of the bowel stumps are comparable, a hand-sewn end-to-end anastomosis may be performed.

The anastomosis is constructed on the anterior wall of the rectum and on the colon as close as possible to the mesenteric taenia. The steps of the hand-sewn anastomosis are as follows:

1. Stay sutures may be placed at the corners of the planned anastomosis to provide traction and facilitate alignment.
2. The posterior approximation layer is started. Beginning at the distal corner, the antimesenteric border of the colonic stump is approximated to the anterior wall of the rectal stump using a continuous 4-0 PDS suture extending along the entire length of the rectum (Fig. 1A).
3. Once adequate approximation is achieved, a colonic and rectal enterotomy are created approximately 1 cm anterior to the posterior layer. The length of each enterotomy is slightly shorter than that of the posterior approximation.
4. The posterior layer of the anastomosis is then constructed. Using a full-thickness or extramucosal 4-0 PDS suture, the rectal and colonic walls are sutured starting from the center of the enterotomies and proceeding towards the right angle, which is crossed (Fig. 1B).
5. Using the same suture (in the case of a double-armed thread), the posterior layer is completed towards the left angle. After crossing the angle, the anterior layer is continued until the two sutures meet in the middle, where they are tied together at the center of the anastomosis. Sutures may be full-thickness or, preferably, extramucosal (Fig. 1C).
6. A second anterior reinforcing layer is then completed using a continuous or interrupted 4-0 PDS suture (Fig. 1D).

A barbed suture may also be used for the construction of the anastomosis. A slowly absorbable barbed suture (3-0, 180-day slower absorbing) may be used for the outer reinforcing layer, whereas a faster-absorbing barbed suture (3-0, 90-day slower absorbing) is preferable for the inner anastomotic layer.

#### Surgical step 4: Anastomotic integrity testing and closure (Video 4)

After completion of the anastomosis, an air–leak test is performed by transanal injection of methylene blue followed by air insufflation.

The mesentery is closed with interrupted sutures to prevent internal herniation. When a wound protector with integrated laparoscopic access is used, a final laparoscopic inspection may be performed to confirm correct bowel positioning and anastomotic configuration. The abdominal wall is then closed in a standard fashion.

## Results

Eleven patients were included in the analysis. The cohort was predominantly male (eight patients, 72.7%), with a median age of 71 years (IQR 51–79) and a median BMI of 22.2 kg/m^2^ (IQR 19.9–23.5). Five patients (45.4%) were classified as ASA III. Recurrent sigmoid volvulus was common, with more than one previous episode documented in 63.6% of patients (nine patients) (Table 1).

**Table 1:** Baseline characteristics of the cohort of patients included in the study

No. of patients	11
Sex	Female: 3 (27.3%)
	Male: 8 (72.7%)
Age (median, IQR)	71 (51–79)
BMI (median, IQR)	22.2 (19.9–23.53)
Tobacco smoking	1 (9.1%)
Anticoagulants	3 (27.3%)
Other chronic medications	8 (72.3%)
Cardiac comorbidity	5 (45.4%)
Respiratory comorbidity	1 (9.1%)
Liver comorbidity	1 (9.1%)
Vascular comorbidity	6 (54.5%)
Immune system comorbidity	1 (9.1%)
Chronic corticosteroid use	1 (9.1%)
Diabetes	2 (18.2%)
Neurological disease	7 (63.6%)
Oncologic disease	-
No. of previous episodes of sigmoid volvulus	Zero = 2 (18.2%)
	One = 2 (18.2%)
	Two = 4 (36.3%)
	Three = 3 (27.3%)
	More than three = 0
ASA (American Society of Anesthesiologists) score	I = 2 (18.2%)
	II = 4 (36.3%)
	III = 5 (45.4%)
	IV = 0
	V = 0
Previous abdominal surgery	1 (9.1%)

*IQR*, interquartile range; *BMI*, body mass index; *ASA*, American Society of Anesthesiologists

All patients presented with abdominal pain and distension, and complete colonic obstruction was observed in ten patients (90.9%). The median time from symptom onset to hospital admission was 3 days (IQR 3–6), and the median interval from admission to surgery was 7 days (IQR 1–8) (Table 2).

**Table 2:** Clinical presentation

No. of patients	11
Abdominal pain	11 (100%)
Abdominal distension	11 (100%)
Nausea/vomiting	3 (27.3%)
Diarrhea	1 (9.1%)
Hematemesis	-
Melena	-
Peritonitis	-
Complete colonic obstruction (flatus/stool)	10 (90.9%)
Time from symptoms occurrence to hospitalization (days) (median, IQR)	3 (3–6)
Time from hospital admission to surgery (days) (median, IQR)	7 (1–8)

*IQR*, interquartile range

Laboratory findings are summarized in Table 3. All patients underwent colonoscopy with successful endoscopic detorsion and decompression. Abdominal CT scan was performed in 72.3% of cases (eight patients).

**Table 3:** Diagnostic parameters

No. of patients	11
White blood cells (WBC) × 10^9^/L (median, IQR)	6.16 (5.5–7.2)
C-reactive protein (CRP) mg/L (median, IQR)	2.4 (1.2–11.5)
Lactate dehydrogenase LDH U/L (median, IQR)	197 (162.5–213.5)
Creatine kinase CK U/L (median, IQR)	52 (40–103.5)
Creatinine mg/dL (median, IQR)	0.72 (0.62–0.98)
Sodium (Na) mmol/L (median, IQR)	139 (139–141)
Potassium (K) mmol/L (median, IQR)	4.0 (3.3–4.4)
Plain abdominal X-Ray	7 (63.6%)
CT scan	8 (72.3%)
Preliminary endoscopic evaluation (colonoscopy)	11 (100%)

*IQR*, interquartile range

Surgical management is detailed in Table 4. Surgery was performed electively (7/11, 63.6%) or as planned during the same admission after endoscopic decompression (early elective, 4/11, 36.4%). The median operative time was 105 min (IQR 90–125). One patient (1/11, 9.1%) underwent an associated procedure (Meckel’s diverticulectomy). No patient required extension of the Pfannenstiel incision or postoperative ICU admission (Table 4).

**Table 4:** Surgical strategy

No. of patients	11
Preliminary endoscopic de-rotation/decompression	11 (100%)
Early elective surgery after endoscopic decompression	4 (36.4%)
Elective surgery	7 (63.6%)
Operative time (minutes) (median, IQR)	105 (90–125)
Associated operation	1 (9.1%)^
Extended Pfannenstiel laparotomy	-
ICU (Intensive Care Unit) admission	-

*IQR*, interquartile range

^Meckel’s diverticulectomy

Postoperative complications occurred in four patients (36.3%), including one Clavien-Dindo grade IIIa event managed non-operatively. Early readmission occurred in three patients (27.3%), all managed without reoperation. No reoperations or postoperative mortality were observed, and all patients were discharged home. The median postoperative length of stay was 6 days (IQR 5–6). Gastrointestinal recovery was prompt, with return of bowel function at a median of 2 days (IQR 2–3) and early resumption of oral intake.

At a median follow-up of 394 days (IQR 246–434), no late complications or recurrence of sigmoid volvulus were observed (Table 5).

**Table 5:** Surgical outcomes

No. of patients	11
Reoperation	-
Complications (total)	4 (36.3%)
Clavien-Dindo I complications	1 (9.1%)
Clavien-Dindo II complications	2 (18.2%)
Clavien-Dindo IIIa complications	1 (9.1%)
Discharge to home	11 (100%)
Discharge to facility	-
Length of postoperative hospital stay (days) (median, IQR)	6 (5–6)
Death	-
Time to nasogastric tube removal (days) (median, IQR)	0 (0–1)
Time to bladder catheter removal (days) (median, IQR)	0 (0–1)
Time to first bowel movement (flatus) (days) (median, IQR)	2 (2–3)
Time to first oral intake (days) (median, IQR)	0 (0–1)
Early recurrence of colonic volvulus	-
30-day hospital readmission	3 (27.3%)
Reason for early hospital readmission	Perianastomotic abscess treated with CT-guided percutaneous drainage (Clavien-Dindo IIIa complication)
	Fever, pneumoperitoneum and pneumonia (Clavien-Dindo II complication)
	Paralytic ileus and hypokalemia (Clavien-Dindo II complication)
Length of follow-up (median, IQR)	394 (246–434)
Follow-up results	Late complications = 0
	Late recurrence of colonic volvulus = 0
	Other = 0

*IQR*, interquartile range

## Discussion

This study reports our initial experience with trans-Pfannenstiel sigmoidectomy for sigmoid volvulus in a consecutive series of 11 patients. Our findings support the technical reliability of the approach and the acceptability of its short-term outcomes in a high-risk cohort. Operative times were limited, no patient required postoperative ICU admission, and no reoperations were needed. Postoperative complications occurred in 36.4% of patients, consistent with rates reported in the literature for sigmoid volvulus surgery [4, 5].

The balance between recurrence prevention and operative risk has been addressed in a recent systematic review and meta-analysis comparing resection and non-resection strategies for sigmoid volvulus. Resection was associated with a marked reduction in recurrence, with a number needed to treat of six, at the cost of increased mortality when all patients were considered [20]. However, when patients with gangrenous sigmoid colon were excluded, resection remained effective in preventing recurrence without a significant increase in mortality. Several studies have shown that recurrent volvulus is associated with increased long-term mortality, particularly when managed with repeated endoscopic decompression alone [10, 21–23]. Mortality in this setting is often related to frailty and baseline functional status rather than to the surgical procedure itself [10, 24]. Moro-Valdezate et al*.* reported lower short-term mortality and improved 2-year overall survival in patients undergoing elective resection compared with conservative management [25]. These data support the concept that repeated conservative management may represent a futile strategy in selected patients who remain candidates for definitive surgery and emphasize the importance of structured frailty assessment in clinical decision-making [26]. The therapeutic pathway adopted in our series aligns with growing evidence supporting planned surgical resection after successful endoscopic decompression. In a retrospective analysis of Medicare beneficiaries aged ≥ 65 years undergoing surgery for sigmoid volvulus after decompression, elective surgery was associated with higher rates of minimally invasive procedures, lower rates of ostomy formation, and greater likelihood of discharge home compared with early elective surgery after successful endoscopic decompression, with similar length of stay [27]. After adjustment for confounders, elective surgery was associated with reduced postoperative morbidity and similar mortality. Similarly, large observational and population-based studies have shown that elective or early elective surgery after decompression sigmoidectomy is associated with lower mortality and improved long-term outcomes compared with emergency surgery or conservative management alone [21, 22, 28, 29]. Surgery performed after initial endoscopic decompression allows patient optimization, including correction of fluid and electrolyte disturbances, nutritional support, and sustained colonic decompression [22, 28]. Conversely, emergency resection has consistently been identified as an independent predictor of postoperative complications and mortality [29, 30].

Other limited open approaches, such as left iliac fossa mini-incisions, have shown satisfactory results [31, 32]. However, techniques based on a limited left lower quadrant incision, including the Sharon procedure, may not ensure adequate visualization of the rectosigmoid junction, with a potential risk of incomplete resection [33].

In contrast, the Pfannenstiel approach provides reliable exposure of the distal sigmoid and upper rectum, facilitates exteriorization of the redundant colon, and allows safe resection while reducing parietal trauma compared with midline laparotomy.

Regarding laparoscopy, the physiological effects of pneumoperitoneum may be poorly tolerated in frail and elderly patients, potentially increasing perioperative risk [34]. In this context, the trans-Pfannenstiel approach represents a pragmatic alternative. It may also be performed under regional anesthesia in selected patients, further reducing the physiological burden of surgery.

In addition to protecting the wound from potential contamination and providing uniform circumferential traction with minimal parietal trauma, modern double-ring wound protectors equipped with an integrated laparoscopic cap (e.g., Alexis®) allow the creation of a temporary laparoscopic working chamber through the Pfannenstiel incision. This may facilitate exploration of the abdominal cavity before proceeding with the resection, verification of mesenteric orientation before and after the anastomosis to avoid unrecognized torsion, and assessment of the anastomosis during the leak test after reconstruction.

In this series, primary colorectal anastomosis was performed in all patients. Although a stapled anastomosis may theoretically be feasible, adequate exteriorization of the bowel to introduce the stapling device may sometimes be technically demanding. Moreover, the caliber and wall thickness of the two bowel ends may differ substantially, particularly between a dilated sigmoid colon and the rectum, potentially limiting optimal compression by stapling devices. In this context, a hand-sewn anastomosis performed under direct visualization at the level of the wound protector allows precise control of each layer and may provide a safer and more adaptable reconstruction.

Evidence from large database analyses and comparative studies suggests that primary anastomosis can be safely performed in selected patients, with outcomes comparable to Hartmann’s procedure [35, 36]. However, advanced age, high ASA grade, repeated endoscopic decompression, and severe frailty have been associated with an increased risk of anastomotic leak [37]. These findings support definitive surgery when feasible and careful patient selection when considering primary anastomosis. In this context, structured frailty assessment should play a key role in guiding surgical decision-making, although it remains underutilized in routine clinical practice [38].

In frail or non-self-sufficient patients, Hartmann’s procedure remains a valid alternative, particularly when the risks associated with an anastomosis outweigh its functional benefits. Alternative non-resective strategies, including endoscopic colopexy and percutaneous endoscopic colostomy, have been proposed for patients deemed unfit for surgery [39–41]. While these approaches may reduce recurrence in selected cases, they do not address the underlying pathology and should be considered palliative options rather than definitive treatments. In our experience, during the interval between emergency admission and surgery, patients underwent preoperative optimization according to ERAS principles. All patients undergoing major abdominal surgery at our institution are managed within ERAS pathways, including adapted protocols for emergency surgery when feasible [42]. Patients are routinely evaluated by the nutritional team and undergo nutritional optimization and prehabilitation when indicated.

This study has several limitations. It represents a single-center experience with a small sample size and no comparator group, as it was designed to assess technical feasibility and safety rather than comparative effectiveness. The technique is based on standard surgical principles and does not require advanced or specialized skills beyond those commonly used in colorectal surgery, which may support its reproducibility.

Some technical limitations of the proposed approach should be acknowledged**.** If the procedure cannot be safely completed through the Pfannenstiel incision, conversion to a laparoscopic approach represents the preferred alternative. When laparoscopy is not feasible, the incision can be further extended to improve exposure. Only in rare cases might a vertical extension resulting in a T-shaped incision be required. Although this scenario has not occurred in our experience, it should nevertheless be acknowledged as a potential limitation of the technique.

## Conclusions

Trans-Pfannenstiel sigmoidectomy is a feasible, safe, and reproducible approach for the surgical management of chronic sigmoid volvulus. When performed after endoscopic decompression in a planned setting, it allows definitive treatment while limiting surgical trauma.

## Supplementary Information

Below is the link to the electronic supplementary material.

ESM 1 (MP4 144 MB)

ESM 2 (MP4 140 MB)

ESM 3 (MP4 152 MB)

ESM 4 (MP4 143 MB)

## Data Availability

The datasets generated and analyzed in this study are not publicly available due to ethical and privacy restrictions but are available from the corresponding author on reasonable request.

## References

[1] Halabi WJ, Jafari MD, Kang CY, Nguyen VQ, Carmichael JC, Mills S, Pigazzi A, Stamos MJ (2014) Colonic volvulus in the United States: trends, outcomes, and predictors of mortality. Ann Surg 259:293–301 23511842 10.1097/SLA.0b013e31828c88ac

[2] Swenson BR, Kwaan MR, Burkart NE, Wang Y, Madoff RD, Rothenberger DA, Melton GB (2012) Colonic volvulus: presentation and management in metropolitan Minnesota, United States. Dis Colon Rectum 55:444–449 22426269 10.1097/DCR.0b013e3182404b3d

[3] Heis HA, Bani-Hani KE, Rabadi DK, Elheis MA, Bani-Hani BK, Mazahreh TS, Bataineh ZA, Al-Zoubi NA, Obeidallah MS (2008) Sigmoid volvulus in the Middle East. World J Surg 32:459–464 18196324 10.1007/s00268-007-9353-3

[4] Oren D, Atamanalp SS, Aydinli B, Yildirgan MI, Başoğlu M, Polat KY, Onbaş O (2007) An algorithm for the management of sigmoid colon volvulus and the safety of primary resection: experience with 827 cases. Dis Colon Rectum 50:489–497 17205203 10.1007/s10350-006-0821-x

[5] Atamanalp SS (2013) Treatment of sigmoid volvulus: a single-center experience of 952 patients over 46.5 years. Tech Coloproctol 17:561–569 23636444 10.1007/s10151-013-1019-6

[6] Bruzzi M, Lefèvre JH, Desaint B, Nion-Larmurier I, Bennis M, Chafai N, Tiret E, Parc Y (2015) Management of acute sigmoid volvulus: short- and long-term results. Colorectal Dis 17:922–928 25808350 10.1111/codi.12959

[7] Alavi K, Poylin V, Davids JS, Patel SV, Felder S, Valente MA, Paquette IM, Feingold DL, Prepared on behalf of the Clinical Practice Guidelines Committee of the American Society of Colon and Rectal Surgeons (2021) The American Society of Colon and Rectal Surgeons clinical practice guidelines for the management of colonic volvulus and acute colonic pseudo-obstruction. Dis Colon Rectum 64:1046–1057 34016826 10.1097/DCR.0000000000002159

[8] Safioleas M, Chatziconstantinou C, Felekouras E, Stamatakos M, Papaconstantinou I, Smirnis A, Safioleas P, Kostakis A (2007) Clinical considerations and therapeutic strategy for sigmoid volvulus in the elderly: a study of 33 cases. World J Gastroenterol 13:921–924 17352024 10.3748/wjg.v13.i6.921 PMC4065930

[9] Yassaie O, Thompson-Fawcett M, Rossaak J (2013) Management of sigmoid volvulus: is early surgery justifiable? ANZ J Surg 83:74–78 22924840 10.1111/j.1445-2197.2012.06182.x

[10] Bock RDE, Vaughan-Shaw PG, Edinburgh Colorectal Group, Clark AJ, Collie M, Collins D, Duff M, Goodbrand S, Mander J, Ventham NT, Paterson HM, Potter MA, Reddy C, Speake D, Din FVN, Dunlop MG, Smith G (2025) Survival outcomes in patients with sigmoid volvulus. Int J Colorectal Dis 40(1). 10.1007/s00384-025-04920-y 40526313 10.1007/s00384-025-04920-y PMC12174257

[11] Tian BWCA, Vigutto G, Tan E, van Goor H, Bendinelli C, Abu-Zidan F, Ivatury R, Sakakushev B, Di Carlo I, Sganga G, Maier RV, Coimbra R, Leppäniemi A, Litvin A, Damaskos D, Broek RT, Biffl W, Di Saverio S, De Simone B, Ceresoli M, Picetti E, Galante J, Tebala GD, Beka SG, Bonavina L, Cui Y, Khan J, Cicuttin E, Amico F, Kenji I, Hecker A, Ansaloni L, Sartelli M, Moore EE, Kluger Y, Testini M, Weber D, Agnoletti V, Angelis ND, Coccolini F, Sall I, Catena F (2023) WSES consensus guidelines on sigmoid volvulus management. World J Emerg Surg 18(1). 10.1186/s13017-023-00502-x 37189134 10.1186/s13017-023-00502-x PMC10186802

[12] Nguyen SH, Tavares K, Chinn A, Russell D, Gillern S, Yheulon C (2022) Is laparoscopy underutilized for sigmoid volvulus? Surg Laparosc Endosc Percutan Tech 32:564–570 35960695 10.1097/SLE.0000000000001074

[13] Agha RA, Sohrabi C, Mathew G, Franchi T, Kerwan A, O’Neill N, PROCESS Group (2020) The PROCESS 2020 guideline: updating consensus preferred reporting of CasESeries in surgery (PROCESS) guidelines. Int J Surg 84:231–235 33189880 10.1016/j.ijsu.2020.11.005

[14] Aljuba YM, Alkadi AT, Hamamdh MG (2024) Segmental thoracic spinal anesthesia for critical patients undergoing abdominal surgeries: a case series and literature review. Cureus 16(11). 10.7759/cureus.74348 39723320 10.7759/cureus.74348 PMC11669299

[15] Uzman S, Donmez T, Erdem VM, Hut A, Yildirim D, Akinci M (2017) Combined spinal-epidural anesthesia in laparoscopic appendectomy: a prospective feasibility study. Ann Surg Treat Res 92:208–213 28382293 10.4174/astr.2017.92.4.208 PMC5378561

[16] Ferrari C, Crippa J, Vailati D, Basta B, Barbaro S, Colasuonno M, Santalucia R, Magistro C (2025) Minimally invasive colorectal surgery under general versus neuraxial anesthesia: a retrospective propensity-score-matched analysis. J Clin Med 14(21). 10.3390/jcm14217684 41227079 10.3390/jcm14217684 PMC12610812

[17] Le Roux JJ, Wakabayashi K, Jooma Z (2023) Emergency awake abdominal surgery under thoracic epidural anaesthesia in a high-risk patient within a resource-limited setting. Cureus 15(2). 10.7759/cureus.34856 36923189 10.7759/cureus.34856 PMC10010061

[18] Romanzi A, Dragani TA, Adorni A, Colombo M, Farro A, Maspero M, Zamburlini B, Vannelli A (2023) Neuraxial anesthesia for abdominal surgery, beyond the pandemic: a feasibility pilot study of 70 patients in a suburban hospital. Updates Surg 75:1691–1697 37278936 10.1007/s13304-023-01554-z PMC10242600

[19] Tavassoli A, Maddah G, Noorshafiee S, Salehi M, Imannezhad S, Ghorbanian E (2016) A novel approach to minimally invasive management of sigmoid volvulus. Acta Med Iran 54:640–643 27888591

[20] Jiang X, Guo S, Yang L (2025) Comparison of recurrence and mortality rates between resection and non-resection surgical methods for treating sigmoid volvulus: a systematic review and meta-analysis. Langenbecks Arch Surg. 10.1007/s00423-025-03952-w 41398319 10.1007/s00423-025-03952-w PMC12799662

[21] Abdelrahim A, Zeidan S, Qulaghassi M, Ali O, Boshnaq M (2022) Dilemma of sigmoid volvulus management. Ann R Coll Surg Engl 104:95–99 34860119 10.1308/rcsann.2021.0123 PMC10335211

[22] Audeguy L, Stella M, Duprez D, Laurent A, Tidadini F, Foote A, Fournier J, Trilling B, Faucheron JL (2025) Sigmoid volvulus: outcomes of surgery and conservative management after initial colonoscopic decompression (the VOLVUCOL study). Br J Surg 112(5). 10.1093/bjs/znaf085 40313075 10.1093/bjs/znaf085

[23] Loria A, Cai X, Gao S, Zhao T, Juviler P, Li Y, Cupertino P, Fleming FJ (2024) Development and validation of multivariable predictive models for recurrence and mortality following nonoperative management of sigmoid volvulus. Colorectal Dis 26:356–363 38151763 10.1111/codi.16849

[24] Ebrahimian S, Lee C, Tran Z, Sakowitz S, Bakhtiyar SS, Verma A, Tillou A, Benharash P, Lee H (2022) Association of frailty with outcomes of resection for colonic volvulus: a national analysis. PLoS One 17(11). 10.1371/journal.pone.0276917 36346811 10.1371/journal.pone.0276917 PMC9642887

[25] Moro-Valdezate D, Martín-Arévalo J, Pla-Martí V, García-Botello S, Izquierdo-Moreno A, Pérez-Santiago L, Pedrós-Giménez JM, Villagrasa R, Peña A, Espí-Macías A (2022) Sigmoid volvulus: outcomes of treatment and predictors of morbidity and mortality. Langenbecks Arch Surg 407:1161–1171 35028738 10.1007/s00423-022-02428-5 PMC9151547

[26] Viswanath M, Clinch D, Ceresoli M, Dhesi J, D’Oria M, De Simone B, Podda M, Di Saverio S, Coccolini F, Sartelli M, Catena F, Moore E, Rangar D, Biffl WL, Damaskos D (2023) Perceptions and practices surrounding the perioperative management of frail emergency surgery patients: a WSES-endorsed cross-sectional qualitative survey. World J Emerg Surg 18(1). 10.1186/s13017-022-00471-7 36653865 10.1186/s13017-022-00471-7 PMC9850554

[27] Hilty Chu B, Loria A, Cai X, Gao S, Dhimal T, Li Y, Cupertino P, Temple LK, Fleming FJ (2024) Comparative analysis of short-term outcomes after semielective and elective surgery for sigmoid volvulus. Surgery 176:1374–1379 39191602 10.1016/j.surg.2024.07.041

[28] Arnold SC, Rafaqat W, Abiad M, Lagazzi E, Hoekman AH, Panossian VS, Nzenwa IC, Paranjape CN, Velmahos GC, Kaafarani HMA, Hwabejire JO (2024) Patience is key: association of surgical timing with clinical outcomes in elderly patients with sigmoid volvulus. Am J Surg 232:81–86 38278705 10.1016/j.amjsurg.2024.01.007

[29] Rasilainen S, Aden M, Kivelä AJ, Pakarinen S, Rintala J, Niemeläinen S, Helavirta I, Moilanen S, Mattila A, Pinta T, Saukkonen K, Vento P, Turkka N, Pengermä P, Häggblom J, Scheinin T (2025) Management and risk factors for colonic volvulus: retrospective national cohort study. BJS Open 9(5). 10.1093/bjsopen/zraf113 40995849 10.1093/bjsopen/zraf113 PMC12461565

[30] Easterday A, Aurit S, Driessen R, Person A, Krishnamurty DM (2020) Perioperative outcomes and predictors of mortality after surgery for sigmoid volvulus. J Surg Res 245:119–126 31415933 10.1016/j.jss.2019.07.038

[31] Seow-En I, Seow-Choen F (2014) Sigmoid volvulus treated by mini-incision. Tech Coloproctol 18:1169–1171 25367827 10.1007/s10151-014-1230-0

[32] Al Dhaheri M, Nada MA, El Ansari W, Kurer M, Ahmed AA (2020) Left iliac fossa mini-incision sigmoidectomy for treatment of sigmoid volvulus. Case series of six patients from Qatar. Int J Surg Case Rep 75:534–538 32950438 10.1016/j.ijscr.2020.09.014 PMC7567052

[33] Inoue K, Yamamoto T, Taniura T, Ishitobi K, Kishimoto A, Kaji S, Tanaka T, Matsubara T, Hidaka M (2026) Surgical management of sigmoid volvulus: a retrospective review of six cases with a focus on the Sharon operation. Surg Case Rep 12. 10.70352/scrj.cr.25-0487 41550110 10.70352/scrj.cr.25-0487 PMC12804092

[34] Lettieri PR, Kunac A, Oliver JB, Anjaria DJ (2022) Sigmoid colectomy for sigmoid volvulus through a limited left lower quadrant transverse laparotomy incision without laparoscopy. Am Surg 88:2233–2234 35505277 10.1177/00031348221093530

[35] Dolejs SC, Guzman MJ, Fajardo AD, Holcomb BK, Robb BW, Waters JA (2018) Contemporary management of sigmoid volvulus. J Gastrointest Surg 22:1404–1411 29569006 10.1007/s11605-018-3747-4

[36] Kazem Shahmoradi M, Khoshdani Farahani P, Sharifian M (2021) Evaluating outcomes of primary anastomosis versus Hartmann’s procedure in sigmoid volvulus: a retrospective-cohort study. Ann Med Surg 62:160–163 10.1016/j.amsu.2021.01.019 PMC7820798 33520215

[37] Tankel J, Gilshtein H, Neymark M, Zuckerman M, Spira R, Yellinek S (2021) Sigmoidectomy following sigmoid volvulus: who is at risk of anastomotic failure? Tech Coloproctol 25:1225–1231 34480672 10.1007/s10151-021-02508-6

[38] Spota A, Cioffi SPB, Altomare M, Kurihara H, Al-Sukhni E, Kaplan LJ, Bass GA (2025) Surgeon attitudes toward risk stratification in emergency surgery for the elderly: an ESTES cross-sectional survey. Eur J Trauma Emerg Surg 51(1):46. 10.1007/s00068-024-02714-5 39853372 10.1007/s00068-024-02714-5

[39] López-Serrano A, Amurrio CA, Hervás J, Latorre P, Ortiz I, Polanco A, Moreno-Osset E (2016) Endoscopic treatment of recurrent sigmoid volvulus with colopexy assisted by t-fasteners and colostomy. Endoscopy 48(Suppl 1):E236–E237. 10.1055/s-0042-109603 27367450 10.1055/s-0042-109603

[40] Jackson S, Hamed MO, Shabbir J (2020) Management of sigmoid volvulus using percutaneous endoscopic colostomy. Ann R Coll Surg Engl 102:654–662 32777932 10.1308/rcsann.2020.0162 PMC7591603

[41] Dahiya DS, Perisetti A, Goyal H, Inamdar S, Singh A, Garg R, Cheng CI, Al-Haddad M, Sanaka MR, Sharma N (2023) Endoscopic versus surgical management for colonic volvulus hospitalizations in the United States. Clin Endosc 56:340–352 37070205 10.5946/ce.2022.166 PMC10244148

[42] Peden CJ, Aggarwal G, Aitken RJ, Anderson ID, Foss BN, Cooper Z, Dhesi JK, French WB, Grant MC, Hammarqvist F, Hare SP, Havens JM, Holena DN, Hübner M, Kim JS, Lees NP, Ljungqvist O, Lobo DN, Mohseni S, Ordoñez CA, Quiney N, Urman RD, Wick E, Wu CL, Young-Fadok T, Scott M (2021) Guidelines for perioperative care for emergency laparotomy enhanced recovery after surgery (ERAS) Society recommendations: Part 1-Preoperative: Diagnosis, rapid assessment and optimization. World J Surg 45:1272–1290 33677649 10.1007/s00268-021-05994-9 PMC8026421

